# Myostatin and IGF-I signaling in end-stage human heart failure: a qRT-PCR study

**DOI:** 10.1186/s12967-014-0365-0

**Published:** 2015-01-16

**Authors:** Júlia Aliz Baán, Zoltán V Varga, Przemyslaw Leszek, Mariusz Kuśmierczyk, Tamás Baranyai, László Dux, Péter Ferdinandy, Thomas Braun, Luca Mendler

**Affiliations:** Department of Biochemistry, Faculty of General Medicine, University of Szeged, Dóm tér 9, H-6720 Szeged, Hungary; Cardiometabolic Research Group, Department of Pharmacology and Pharmacotherapy, Semmelweis University, Nagyvárad tér 4, H-1089 Budapest, Hungary; Institute of Cardiology, ul. Alpejska 42, 04-628 Warszawa, Poland; Pharmahungary Group, Szeged, Hungary; Department I - Cardiac Development and Remodelling, Max Planck Institute for Heart and Lung Research, Ludwigstrasse 43, D-61231 Bad Nauheim, Germany; Institute of Biochemistry II, Goethe University, Faculty of Medicine, Theodor-Stern-Kai 7, 60590 Frankfurt, Germany

**Keywords:** Heart failure, Myostatin, IGF-I, Activin receptor IIB, IGF-I receptor, qRT-PCR, microRNA-208, miRNA

## Abstract

**Background:**

Myostatin (Mstn) is a key regulator of heart metabolism and cardiomyocyte growth interacting tightly with insulin-like growth factor I (IGF-I) under physiological conditions. The pathological role of Mstn has also been suggested since Mstn protein was shown to be upregulated in the myocardium of end-stage heart failure. However, no data are available about the regulation of gene expression of Mstn and IGF-I in different regions of healthy or pathologic human hearts, although they both might play a crucial role in the pathomechanism of heart failure.

**Methods:**

In the present study, heart samples were collected from left ventricles, septum and right ventricles of control healthy individuals as well as from failing hearts of dilated (DCM) or ischemic cardiomyopathic (ICM) patients. A comprehensive qRT-PCR analysis of Mstn and IGF-I signaling was carried out by measuring expression of Mstn, its receptor Activin receptor IIB (ActRIIB), IGF-I, IGF-I receptor (IGF-IR), and the negative regulator of Mstn miR-208, respectively. Moreover, we combined the measured transcript levels and created complex parameters characterizing either Mstn- or IGF-I signaling in the different regions of healthy or failing hearts.

**Results:**

We have found that in healthy control hearts, the ratio of Mstn/IGF-I signaling was significantly higher in the left ventricle/septum than in the right ventricle. Moreover, Mstn transcript levels were significantly upregulated in all heart regions of DCM but not ICM patients. However, the ratio of Mstn/IGF-I signaling remained increased in the left ventricle/septum compared to the right ventricle of DCM patients (similarly to the healthy hearts). In contrast, in ICM hearts significant transcript changes were detected mainly in IGF-I signaling. In paralell with these results miR-208 showed mild upregulation in the left ventricle of both DCM and ICM hearts.

**Conclusions:**

This is the first demonstration of a spatial asymmetry in the expression pattern of Mstn/IGF-I in healthy hearts, which is likely to play a role in the different growth regulation of left vs. right ventricle. Moreover, we identified Mstn as a massively regulated gene in DCM but not in ICM as part of possible compensatory mechanisms in the failing heart.

## Background

Myostatin (Mstn), the growth inhibitor of skeletal muscle [1], was shown to be expressed in the heart tissue [2] with controversial data about its role in myocardial physiology and pathophysiology. Based on the analysis of constitutive Mstn knockout mice, numerous studies have demonstrated that Mstn inhibits cardiac growth and contractility and induces fibrosis [3-6]. Nevertheless, by analyzing adult cardiac-specific Mstn mutants we have revealed recently a beneficial role of Mstn in maintaining cardiac energy homeostasis and preventing pathological cardiac hypertrophy [7].

Insulin-like growth factor I (IGF-I), on the other hand was shown to play a pivotal role in cardiovascular physiology and aging [8-10]. In concert with insulin itself, IGF-I proved to be a positive regulator of cardiac growth and contractility under both physiological and pathological conditions [11-17]. Previous *in vitro* studies described a tight interplay between Mstn and IGF-I [3,18-20] and proposed that Mstn might be a cardiac chalone of IGF-I since cardiac growth induced by IGF-I was feed-backed by the overexpression of the negative growth regulator Mstn [21]. However, no studies have systematically analyzed the relevance of their possible reciprocal regulation at the gene expression level in healthy or failing human hearts. Previous investigations focused only on Mstn protein activation that has been shown to be accelerated in hearts of dilated or ischemic cardiomyopathic patients (DCM or ICM, respectively) [22]. Moreover, no data exist in the literature about the expression pattern of Mstn in comparison with IGF-I and their receptors in various regions (i. e. left ventricles (LV) versus right ventricles (RV)) of the human heart. Given the different functional requirements LV and RV should cope with, and the markedly different development of these regions, one could assume that the gene expression pattern of Mstn and IGF-I signaling might show remarkable spatial differences under both physiological and pathological conditions. In the present qRT-PCR study we report that Mstn/IGF-I signaling differs in LV versus RV even in healthy hearts and shows significant differences in DCM versus ICM patients.

## Methods

### Study design

All procedures followed were in accordance with the ethical standards of the responsible committee on human experimentation (institutional and national) and with the Helsinki Declaration of 1975. Informed consent was obtained from all patients for being included in the study according to the protocol approved by the Local Ethics Committee (IK-NP-0021-24/1426/14). Healthy human hearts were obtained from organ donor patients (CONT, n = 5) whose hearts were explanted but due to technical reasons (CMV infection, extensive damage during harvest and size donor/recipient mismatch), not used for transplantation. The donors did not present any important previous medical history or any abnormalities in ECG and echocardiography (LV dimensions/contractility within normal ranges); these organ donors had died from head trauma, cerebral or subarachnoid hemorrhage. Explanted end-stage failing hearts were obtained from patients with advanced heart failure of non-ischaemic (DCM) (n = 5) or ischaemic aethiology (ICM) (n = 5). Before transplantation the clinical state of all patients was determined according to the New York Heart Association (NYHA) classification; patients of NYHA class III–IV underwent a clinical assessment that included resting electrocardiogram, echocardiography and hemodynamic measurements.

### Preparation of cardiac tissue

Tissue samples of the right and left ventricular free walls and the inter-ventricular septum were taken at the time of explantation (avoiding scarred, fibrotic, or adipose tissue, endocardium, epicardium or coronary vessels). The samples were rinsed immediately, blotted dry, frozen in liquid nitrogen and kept at − 80°C until further processing.

### qRT-PCR analysis of mRNA transcripts

Total RNA was isolated from the LV and RV as well as from inter-ventricular septum (S) of the CONT, DCM or ICM patients with the guanidinium thiocyanate-phenol-chloroform method [23], followed by reverse transcription (Sigma MMLV- Moloney Murine Leukemia Virus Reverse Transcriptase, 28025-013). For the detection of the transcript levels of myostatin (Mstn), activin receptor IIB (ActRIIB), insulin-like growth factor I (IGF-I) and insulin-like growth factor I receptor (IGF-IR) quantitative PCR was carried out with SYBR GREEN master mix (Fermentas) on a Light Cycler 1.5 (Roche Applied Science). Since some of the genes generally used for internal normalization in qRT-PCR contain several pseudogenes (e.g. GAPDH, beta-actin) of which co-amplification may compromise their reliability as reference genes [24], hipoxanthine-guanine phosphorybosyltransferase (HPRT) has been used as a single internal control gene in our experiments. Indeed, HPRT expression did not significantly change between different groups or heart regions (data not shown). Cycle conditions were set as an initial denaturation step for 10 min at 95°C, followed by 45 cycles of 10 sec at 95°C for template denaturation, 10 sec for annealing phase at 58°C and 10 sec at 72°C for extension. Specificity of the PCR products was confirmed by melting curve analysis followed by the verification of the amplicon length on 1.5% agarose gels stained by ethidium bromide. Primer pairs for Mstn, ActRIIB, IGF-I, IGF-IR and HPRT were designed to intron spanning exons by Primer 3 Input (version 0.4.0) software and tested to avoid primer dimers, unspecific amplification and self-priming formation (Table 1).

**Table 1:** **Primer properties used in qRT-PCR**

**Target**	**Accession number**	**Forward primer**	**Reverse primer**	**Efficiency**	**Product size (bp)**
Mstn	NM_005259.2	TTCGTCTGGAAACAGCTCCT	CATTTGGGTTTTCCATCCAC	1.783	220
ActRIIB	NM_001106.3	TGACTTTGGCTTGGCTGTTC	ATGTACTCATCCACGGGTCC	1.834	219
IGF-I	XM_005268835.1	ATGCTCTTCAGTTCGTGTGTG	GGGTCTTGGGCATGTCGGTG	1.758	219
IGFI-R	NM_000875.3	GACAACCAGAACTTGCAGCA	GATTCTTCGACGTGGTGGTG	1.714	241
HPRT	NM_000194.2	TGCTCGAGATGTGATGAAGG	TCCCCTGTTGACTGGTCATT	2.044	192

bp: base pair, Mstn: myostatin, ActRIIB: activin receptor IIB, IGF-I: insulin-like growth factor I, IGFI-R: insulin-like growth factor I receptor, HPRT: hypoxanthine-guanine phosphorybosyltransferase.

### qRT-PCR analysis of miRNA transcripts

From the above detailed total RNA isolates, cDNA was synthetized and quantitative real-time PCR was performed with miRCURY LNA™ Universal RT microRNA PCR kit (Exiqon, Denmark) on LightCycler®480 (Roche, Switzerland) according to the manufacturer’s instructions. Briefly, total RNA was diluted to a final concentration of 5 ng/μL, and mixed with Reaction buffer and Enzyme mix provided with the kit in a final volume of 10 μL. Reaction mixture was incubated for 60 min at 42°C, and reverse transcriptase was heat-inactivated for 5 min at 95°C. Then, cDNA was diluted to 80x and 4 μL of diluted cDNA were mixed with 6 μL of the PCR Master mix and PCR primer mix supplied by the manufacturer. The primers for both microRNA-208b and microRNA-103a-3p was designed and prepared by using Exiqon’s LNA™ technology. Polymerase was activated for 10 min at 95°C, and microRNA-208b and microRNA-103a-3p were amplified and quantified (denaturation: 10 sec at 95°C; annealing/synthesis: 1 min at 60°C). The specificity of amplifications was assessed by melting curve analysis (42°C to 80°C) and by agarose gel electrophoresis (1%). At last, crossing point values (Cp) were calculated. MicroRNA-208b Cp values were normalized to the corresponding housekeeping microRNA-103a-3p Cp values. Then, all pairwise ΔCp value normalization was carried out to control ΔCp values, and expressed as mean of the three replicated of 2-ΔΔCp values (fold change).

### Definition of parameters characterizing Mstn and IGF-I signaling in the heart

In order to describe the additive effect of Mstn and its receptor ActRIIB transcript levels we combined these data and defined a multiplied value of Mstn x ActRIIB as ‘Mstn signaling index’ for each analyzed sample. Similarly, we created a multiplied value of IGF-I x IGF-IR transcript levels referred to as ‘IGF-I signaling index’. To show the ratio of Mstn and IGF-I signaling in the different regions of healthy or failing hearts we divided the measured transcript levels by each other to produce either growth factor ratio of Mstn/IGF-I or receptor ratio of ActRIIB/IGF-IR. Finally, we created a complex parameter characterizing the combined ratio of Mstn/IGF-I signaling by dividing ‘Mstn signaling index’ (Mstn x ActRIIB) by ‘IGF-I signaling index’ (IGF-I x IGF-IR) referred to as ‘Mstn/IGF-I signaling index’ ((Mstn x ActRIIB)/(IGF-I x IGF-IR)).

### Statistical analysis

Statistical analysis was performed by one-way/two-way ANOVA or non-parametric *t*-test (Welch test) using Prism software (GraphPad Software, Inc.; San Diego, CA), as appropriate. All data were expressed as means ± SEM. For all analyses, a P value <0.05 was considered statistically significant. The individual P-values are indicated in the figure legends.

## Results

### Study patients

A detailed summary of the pre-transplant data and drug therapy of study subjects are shown in Table 2. Both female and male patients were included in all groups. The age of ICM patients differed as expected significantly from both CONT and DCM, since ICM patients are usually diagnosed with end-stage heart failure later than DCM patients. DCM and ICM patients were in either NYHA class III or IV with no difference in pulmonary artery pressure (PAP, PWP) left ventricle size parameters (LVED, LVSD, IVS, PW) or left ventricular ejection fraction (LVEF) among groups. Extra care was taken to exclude diabetic (insulin-treated) patients from the study to avoid possible modification of the IGF-I signaling by insulin treatment. All patients were managed with angiotensin-converting enzyme (ACE)-inhibitors, beta-blockers and diuretics, however, aspirin and statins were only used in case of ICM patients. CONT subjects received iv. treatment composed of very low catecholamine infusion (noradrenaline: 01.-0.2 μg/kg/min, dopamine: 1-2 μg/kg/min) whereas adequate fluid balance was maintained with intravenous fluids including colloids (e.g. Voluven - hydroxyethyl starch) and Desmopressin.

**Table 2:** **Clinical, echocardiographic and hemodynamic characteristics of DCM and ICM patients**

	**CONT**	**DCM**	**ICM**
Number of samples	5	5	5
Gender (female/male)	3/2	2/3	4/1
Age (year)	29 ± 9	39 ± 10	57 ± 11^*#^
NYHA functional class III/IV, *n*	n.a.	0/5	3/2
PAP, mmHg	n.a.	31.6 ± 4.7	30.8 ± 5.6
PWP, mmHg	n.a.	24 ± 4.3	21 ± 3.5
LVED, mm	n.a.	68 ± 4	71 ± 4
LVSD, mm	n.a.	63 ± 5	61 ± 8
PW, mm	n.a.	9.5 ± 0.5	10 ± 1.5
IVS, mm	n.a.	10 ± 0.7	11 ± 1.5
LVEF, %	n.a.	16 ± 3	23 ± 3
**Medications**			
ACE-inhibitor	-	++	++
β-Blocker	-	++	++
Diuretics	-	++	++
Digitalis	-	+	+
PDE-inhibitor	-	++	++
Dopamine/Noradrenaline	++	++	+
Statin	-	-	++
Aspirin	-	-	++
Desmopressin	++	-	-

Values are given in mean ± SEM; *p < 0.05 compared to control; ^#^p < 0.05 compared to DCM Abbreviations: CONT: healthy control individuals, DCM: dilated cardiomyopathy, ICM: ischemic cardiomyopathy, NYHA: New York Heart Association, PAP: mean pulmonary artery pressure, PWP: mean pulmonary wedge pressure, LVED: left ventricular end-diastolic diameter, LVSD: left ventricular end-systolic diameter, PW: posterior wall thickness, IVS: interventricular septum thickness, LVEF: left ventricular ejection fraction, ACE: angiotensin converting enzyme, PDE: phosphodiesterase, n.a.: not applicable.

### Mstn and IGF-I signaling in healthy control hearts

**Figure 1:**
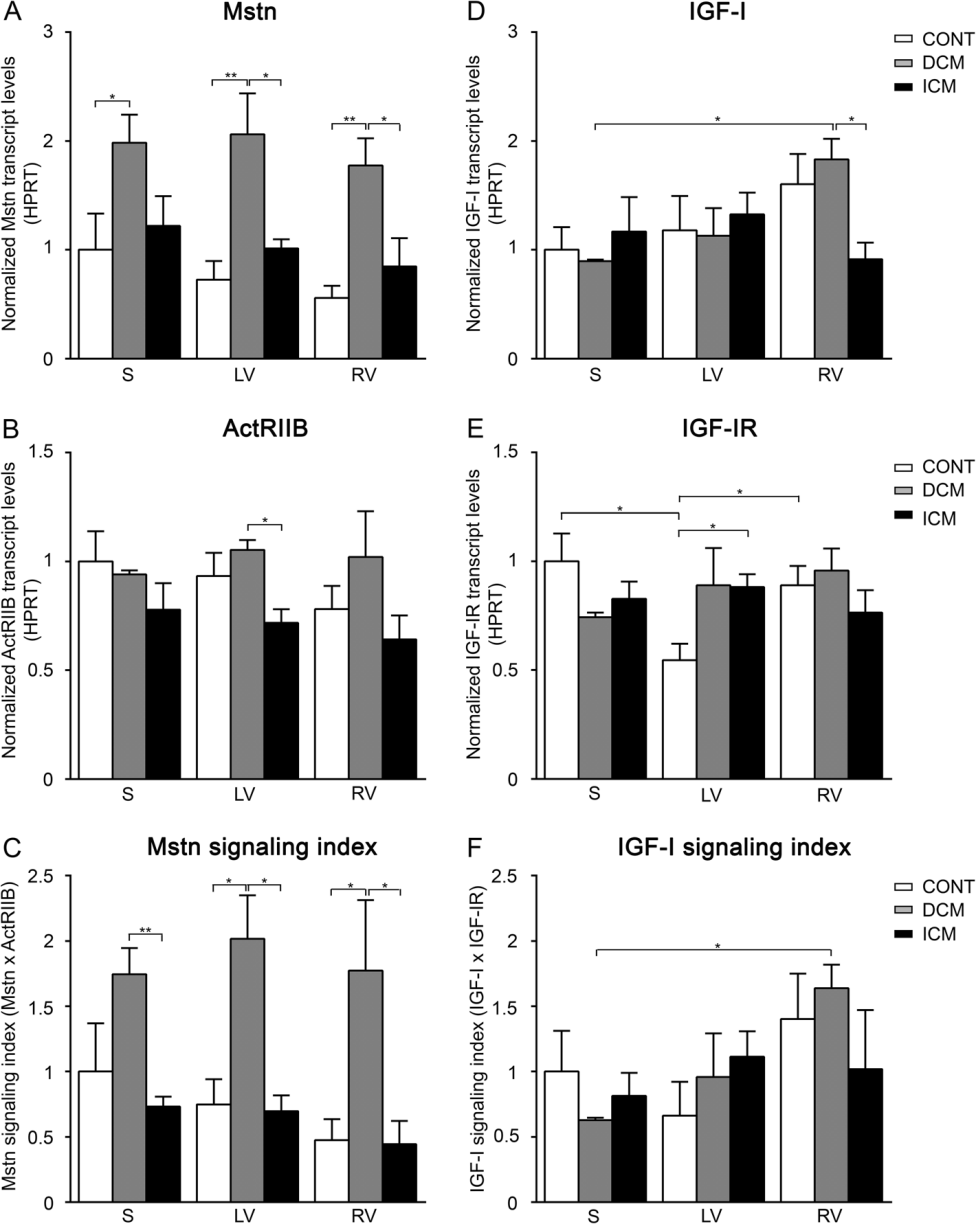
**Gene expression levels of Mstn and IGF-I as well as their receptors ActRIIB and IGF-IR normalized to HPRT in the septum (S), left ventricles (LV) and right ventricles (RV) of control (CONT)-, DCM-, and ICM hearts, respectively.** Bars represent normalized Mstn- **(A)**, ActRIIB- **(B)**, IGF-I- **(D)**, and IGF-IR- **(E)** transcript levels. Panel **C** shows ‘Mstn signaling index’ (Mstn multiplied by ActRIIB mRNA levels) while panel **F** represents ‘IGF-I signaling index’ (IGF-I multiplied by IGF-IR mRNA levels). Data are expressed in mean ± SEM, asterisks show significant differences (n = 5, *p < 0.05, **p < 0.01).

**Figure 2:**
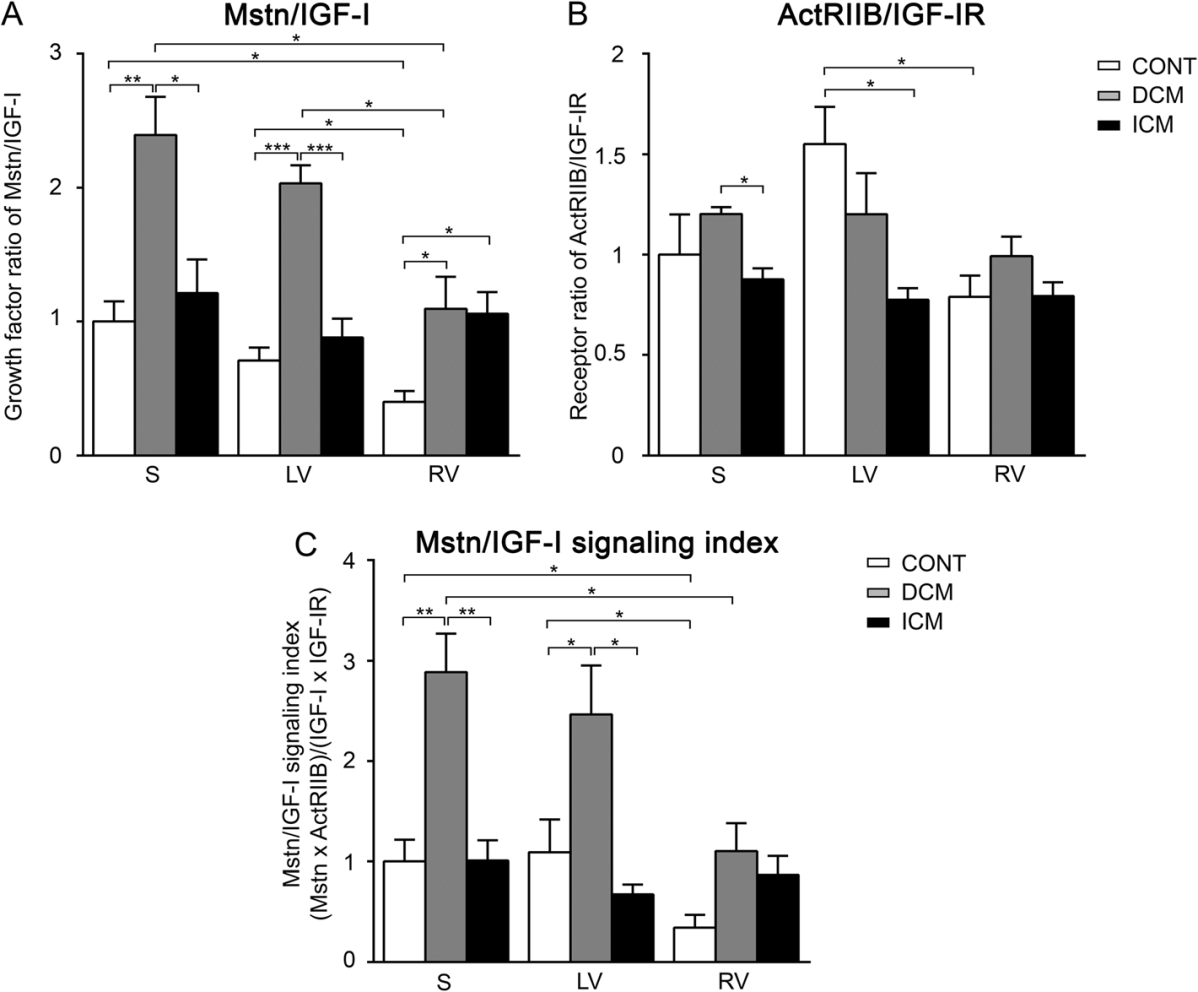
**Growth factor ratio of Mstn/IGF-I (A), receptor ratio of ActRIIB/IGF-IR (B) and ‘Mstn/IGF-I signaling index’ (C) in the septum (S), left ventricles (LV) and right ventricles (RV) of control (CONT)-, DCM-, and ICM hearts, respectively.** Panel **C** represents the ratio of Mstn and IGF-I signaling referred to as ‘Mstn/IGF-I signaling index’ (‘Mstn signaling index’ divided by ‘IGF-I signaling index’ (Mstn x ActRIIB)/(IGF-I x IGF-IR)). Data are expressed in mean ± SEM, asterisks show significant differences (n = 5, *p < 0.05, **p < 0.01, ***p < 0.001).

So far, no comprehensive study has been carried out to reveal gene expression of the growth regulators Mstn and IGF-I and their receptors in different regions of healthy human hearts. In the present work we detected no significant difference in Mstn (Figure 1A) and ActRIIB (Figure 1B) transcript levels nor in Mstn signaling index (Figure 1C) between septum (S), left ventricle (LV) and right ventricle (RV), however, Mstn levels showed decreasing tendency in the RV (Figure 1A). In contrast, both IGF-I mRNAs (Figure 1D) and IGF-I signaling index (Figure 1F) followed an increasing tendency in RV accompanied with a significantly higher IGF-IR levels compared to those of LV (Figure 1E). As a consequence, the ratio of Mstn/IGF-I gene expression (Figure 2A) as well as those of ActRIIB/IGF-I receptors (Figure 2B) and finally the ratio of Mstn to IGF-I signaling (Figure 2C) all showed significantly higher values in LV/S as compared to RV. These data clearly demonstrate that Mstn signaling dominates over IGF-I in the LV more than in RV of healthy human hearts.

### Mstn and IGF-I signaling in DCM patients compared to healthy controls

In DCM patients we measured massive upregulation of Mstn mRNA (Figure 1A) associated with an increased Mstn signaling index (Figure 1C) in all heart regions compared to CONT, although ActRIIB levels remained relatively unchanged (Figure 1B). Similar to healthy hearts, we found a significant upregulation of IGF-I transcripts (Figure 1D) as well as of IGF-I signaling index (Figure 1F) in RV of the failing hearts when compared to those of S, although IGF-IR expression did not show significant difference in either region nor in comparison to CONT hearts (Figure 1E). Thus, the ratio of Mstn/IGF-I mRNA levels (Figure 2A) and the Mstn/IGF-I signaling index (Figure 2C) proved to be significantly higher in the left versus right side of the DCM hearts and showed much higher levels than those of the CONT regions. Since the ratio of the ActRIIB/IGF-I receptors (Figure 2B) did not change significantly we can conclude that Mstn was upregulated in all regions of failing hearts in DCM patients as compared to CONT. However, given the higher IGF-I levels in RV, left and right side of failing heart differed significantly from each other in regard to the ratio of Mstn/IGF-I signaling similar to those of healthy ones (Figure 2C).

### Mstn and IGF-I signaling in ICM patients compared to healthy controls

In contrast to DCM patients, we could not detect any difference in either Mstn (Figure 1A) or ActRIIB transcript levels (Figure 1B) or in Mstn signaling index (Figure 1C) in any heart region of ICM patients compared to those of CONT. Similarly, IGF-I (Figure 1D) and IGF-IR levels (Figure 1E) as well as IGF-I signaling index (Figure 1F) did not differ in ICM heart regions; however, in comparison to CONT IGF-I showed a decreasing tendency of expression in the RV, while increased expression of IGF-IR in the LV was present. Consequently, both ratios of Mstn/IGF-I (Figure 2A) and ActRIIB/IGF-IR (Figure 2B) were similar in all analyzed regions of ICM hearts. However, significantly higher Mstn/IGF-I ratios were revealed in RV due to decreased IGF-I levels as well as significantly lower ActRIIB/IGF-IR ratios in LV when compared to CONT hearts (due to increased IGF-IR levels). In summary, ICM hearts did not show significantly altered modulation of Mstn signaling in either heart region, whereas IGF-I signaling, in contrast to the healthy situation, seemed to be moderately induced in the LV, while inhibited in the RV.

### Differences in Mstn/IGF-I signaling between DCM and ICM patients

Based on our results, all regions of DCM hearts showed significantly higher Mstn levels (Figure 1A) as well as elevated Mstn signaling index (Figure 1C) than those of ICM hearts. Moreover, ActRIIB (Figure 1B) also revealed increased levels in LV of DCM vs. ICM patients. Nevertheless, we found no significant difference in IGF-I signaling on the left side of failing hearts (Figure 1D-F), although significantly less IGF-I transcripts were evident on the right side of ICM hearts in comparison with that of DCM ones (Figure 1D). As a consequence, all parameters describing the ratio of Mstn to IGF-I signaling (Figure 2A-C) showed significantly increased values in the LV of DCM versus ICM hearts. Although in the RV we have revealed similar signaling ratio in both types of failing hearts (Figure 2A-C) the reason for that was an upregulation of Mstn signaling in DCM patients while a downregulation of IGF-I signaling in ICM heart samples.

### miR-208 expression in relation to Mstn expression in DCM and ICM patients compared to healthy controls

In parallel with the massive upregulation of Mstn mRNA in the left ventricle of DCM patients we measured a mild upregulation of miR-208b (1.505 fold change) compared to CONT. A similar but less pronounced upregulation was seen in ICM hearts (1.405 fold change) when compared to CONT, however, no significant difference was detected between DCM and ICM patients.

## Discussion

**Figure 3:**
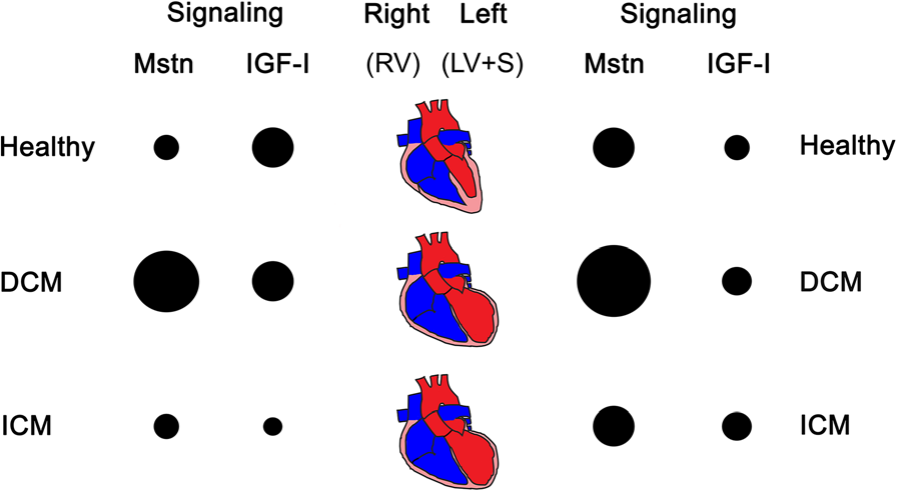
**Summary of gene expression alterations regarding myostatin (Mstn) and IGF-I signaling in healthy human hearts as well as in DCM and ICM patients, respectively.** Bigger circles represent higher expression of the signaling molecules in control vs. DCM or ICM hearts or in left (left ventricle (LV) + septum (S)) vs. right (right ventricle (RV)) side of the hearts.

In the present comprehensive qRT-PCR study we have found that Mstn dominated over IGF-I signaling much more in the LV than in the RV of healthy human hearts, and that DCM hearts upregulated Mstn expression in contrast to ICM hearts. This is the first demonstration that Mstn/IGF-I signaling differs in LV and RV in healthy hearts and shows significant alterations in end-stage heart failure due to DCM and ICM (Figure 3).

Several studies exist in the literature, indicating the presence and/or de-regulation of both Mstn and IGF-I under different conditions in the heart tissue, however, the majority of data were collected from whole hearts or separately from the LV [2,11,12,22]. Regarding Mstn expression in healthy hearts, only one study has been published so far on higher transcript levels of Mstn in LV as compared to RV in young piglet hearts [25]. In line with these data we have demonstrated here an obvious reciprocal regulation of Mstn and IGF-I in LV compared to RV characterized by elevated ratios of both Mstn/IGF-I and ActRIIB/IGF-IR transcripts in healthy human LV (Figure 3). Septum (S) samples, as being part of LV from the functional point of view, revealed similar values to those of LV in most cases. We assume that cardiomyocyte growth in LV/S should be balanced more tightly by growth inhibitors (i.e. Mstn) than that of RV since LV is exposed to higher-pressure overload, while RV to a relatively higher-volume overload. In addition to Mstn and IGF-I, several other factors have been reported to be enriched in either LV or RV; their asymmetric expression might reflect a molecular predisposition of myocardium to LV-concentric and RV-eccentric remodeling during postnatal development [25,26]. Similarly, higher expression levels in LV vs. RV have been demonstrated for cytochrome c oxidases and PGC1α, both of which are known to contribute to maintain oxidative metabolism [27]. Recently, we have also shown that Mstn plays an important role in the regulation of oxidative metabolism of the myocardium [7]. Therefore our results support the idea that the elevated ratio of Mstn/IGF-I signaling is an important regulatory mechanism under physiological conditions maintaining higher workload and oxidative metabolism in the LV.

In human failing hearts (in whole hearts or LV), Mstn protein activation was reported to be accelerated in both DCM and ICM patients [22]. In parallel with this observation several groups found increased serum levels of Mstn protein in patients suffering from heart failure, although no correlation was demonstrated with the severity or type of cardiac disease [22,28-30]. One should also consider that elevated serum myostatin levels in cardiomyopathic patients might be a combined effect of increased secretion from both cardiac and skeletal muscles, although exercise training lead to a reduction of myostatin levels only in skeletal muscles but not in serum of patients with chronic heart failure [31]. Nevertheless, it has been not clarified, whether protein activation in failing heart is regulated either at the level of gene expression or posttranscriptionally. Here we show that DCM hearts are indeed characterized by upregulated Mstn transcripts in both LV/S and RV supporting the previous reports on protein activation in LV [22]. However, we could not detect any significant elevation of Mstn transcript levels in ICM patients (Figure 3). We also tested the miRNA-dependent posttranscriptional regulation of Mstn. MiR-208 have been reported to be a negative regulator of Mstn expression [32], and to be upregulated in various forms of cardiomyopathy [33,34] and myocardial ischemia [35]. In line with these we detected a mild upregulation of miR-208b in the LV of DCM patients characterized by massive increase of myostatin transcripts that might suggest an adaptive counter regulatory mechanism fine-tuning the expression of Mstn during heart failure. Since we have found no difference in miR-208b expression between DCM and ICM hearts, it is likely that higher levels of myostatin mRNAs are mainly regulated at the level of transcription.

Although alterations in Mstn gene expression are followed by similar changes at the protein level in most cases [36-38], the (extracellular) promyostatin-pool might be posttranslationally activated by the cleavage of the propeptide [39-41]. Thus, ICM patients might have a Mstn activation at the protein level [22], however, not at the level of gene expression, whereas DCM hearts react with significant upregulation of Mstn transcripts. Similar to the results of George et al. [22] we detected lower level of ActRIIB in ICM than in DCM patients, but this occured only in the LV. On the other hand, IGF-I expression was shown to be dynamically regulated in the course of heart failure as an important compensatory mechanism; however, conflicting data exist in the literature with both down- and upregulated IGF-I levels in end-stage heart failure [11,14,15]. We could confirm significant decrease of IGF-I signaling in the RV of ICM patients but not in LV or in DCM patients. Moreover, DCM patients still maintained the physiological difference in Mstn/IGF-I signaling ratios in LV vs. RV, whereas no asymmetric gene expression pattern was detected in ICM patients (Figure 3). The mechanisms leading to different regulation of growth factor signaling in DCM and ICM patients remain to be clarified, however, it might relate to the different pathomechanism of heart failure and/or alternative regulation of compensatory mechanisms. Indeed, Mstn upregulation detected in DCM hearts might be part of the compensation reactions since we have reported recently that acute cardiac-specific deletion of Mstn in adult mouse hearts induces dilated cardiomyopathy followed by a massive compensatory up-regulation of Mstn in non-cardiomyocytes [7]. It is known, however, that various cardiac disease conditions, i.e. hypoxia can result in an imbalance of chamber-associated gene expression in myocardium [25-27,42]. Therefore, ICM hearts of ischemic origin might not be able to compensate as effective as do DCM patients by upregulating Mstn to maintain oxidative metabolism [7] and by regulating their IGF-I signaling to counter-act the decreased contractility [11]. However, further research is needed to elucidate the physiological and pathological relevance of the complex Mstn/IGF-I network in human heart.

## Conclusions

Altogether, our results uncovered a spatial asymmetry in the expression pattern of Mstn/IGF-I in healthy hearts, which is likely to play a role in the different growth regulation of LV vs. RV. Moreover, we identified Mstn as a massively regulated gene in DCM but not in ICM as part of possible compensatory mechanisms in the failing heart.

### Limitations

A clear limitation of the study is that our conclusions are only based on transcript data. However, our goal was to clarify transcriptional and posttranscriptional regulation of myostatin/IGF-1 signaling which is missing from the literature and not the recapitulation of other studies mainly concentrating on protein changes in heart failure patients [22,28-30].

## Abbreviations

Mstn: Myostatin
ActRIIB: Activin receptor IIB
IGF-I: Insulin-like growth factor I
IGF-IR: Insulin-like growth factor I receptor
HPRT: Hypoxanthine guanine phosphorybosyl-transferase
DCM: Dilated cardiomyopathy
ICM: Ischemic cardiomyopathy
CONT: Control
CMV: Cytomegalovirus
miR: microRNA
NYHA: New York Heart Association
PAP: Mean pulmonary artery pressure
PWP: Mean pulmonary wedge pressure
LVED: Left ventricular end-diastolic diameter
LVSD: Left ventricular end-systolic diameter
PW: Posterior wall thickness
IVS: Interventricular septum thickness
LVEF: Left ventricular ejection fraction
ACE: Angiotensin converting enzyme
PDE: Phosphodiesterase
PGC1α: Peroxisome proliferator-activated receptor-γ coactivator 1-α

## References

[1] McPherron AC, Lawler AM, Lee SJ (1997). Regulation of skeletal muscle mass in mice by a new TGF-beta superfamily member. Nature.

[2] Sharma M, Kambadur R, Matthews KG, Somers WG, Devlin GP, Conaglen JV, Fowke PJ, Bass JJ (1999). Myostatin, a transforming growth factor-ß superfamily member, is expressed in heart muscle and is upregulated in cardiomyocytes after infarct. J Cell Physiol.

[3] Morissette MR, Cook SA, Foo S, McKoy G, Ashida N, Novikov M, Scherrer-Crosbie M, Li L, Matsui T, Brooks G, Rosenzweig A (2006). Myostatin regulates cardiomyocyte growth through modulation of Akt signaling. Circ Res.

[4] Morissette MR, Stricker JC, Rosenberg MA, Buranasombati C, Levitan EB, Mittleman MA, Rosenzweig A (2009). Effects of myostatin deletion in aging mice. Aging Cell.

[5] Artaza JN, Singh R, Ferrini MG, Braga M, Tsao J, Gonzales-Cadavid NF (2008). Myostatin promotes a fibrotic phenotypic switch in multipotent C3H 10T1/2 cells without affecting their differentiation into myofibroblasts. J Endocrinol.

[6] Rodgers BD, Interlichia JP, Garikipati DK, Mamidi R, Chandra M, Nelson OL, Murry CE, Santana LF (2009). Myostatin represses physiological hypertrophy of the heart and excitation-contration coupling. J Physiol.

[7] Biesemann N, Mendler L, Wietelmann A, Hermann S, Schäfers M, Krüger M, Boettger T, Borchardt T, Braun T (2014). Myostatin regulates energy homeostasis in the heart and prevents heart failure. Circ Res.

[8] Ungvari Z, Csiszar A (2012). The emerging role of IGF-1 deficiency in cardiovascular aging: recent advances. J Gerontol A Biol Sci Med Sci.

[9] Bailey-Downs LC, Sosnowska D, Toth P, Mitschelen M, Gautam T, Henthorn JC, Ballabh P, Koller A, Farley JA, Sonntag WE, Csiszar A, Ungvari Z (2012). Growth hormone and IGF-1 deficiency exacerbate high-fat diet-induced endothelial impairment in obese Lewis dwarf rats: implications for vascular aging. J Gerontol A Biol Sci Med Sci.

[10] Toth P, Tucsek Z, Tarantini S, Sosnowska D, Gautam T, Mitschelen M, Koller A, Sonntag WE, Csiszar A, Ungvari Z. IGF-1 deficiency impairs cerebral myogenic autoregulation in hypertensive mice. J Cereb Blood Flow Metab 2014, doi:10.1038/jcbfm.2014.156. 10.1038/jcbfm.2014.156 PMC4269740 25248835

[11] Serneri GG, Modesti PA, Boddi M, Cecioni I, Pannicia R, Coppo M, Galanti G, Simonetti I, Vanni S, Papa L, Bandinelli B, Migliorini A, Modesti A, Maccherini M, Sani G, Toscano M (1999). Cardiac growth factors in human hypertrophy: relations with myocardial contractility and wall stress. Circ Res.

[12] Serneri GG, Boddi M, Cecioni I, Vanni S, Coppo M, Papa ML, Bandinelli B, Bertolozzi I, Polidori G, Toscano T, Maccherini M, Modesti PA (2001). Cardiac angiotensin II formation in the clinical course of heart failure and its relationship with left ventricular function. Circ Res.

[13] Palmieri EA, Benincasa G, Di Rella F, Casaburi C, Monti MG, De Simone G, Chiariotti L, Palombini L, Bruni CB, Sacca L, Cittadini A (2002). Differential expression of TNF-alpha, IL-6, and IGF-1 by graded mechanical stress in normal rat myocardium. Am J. Physiol Heart Circ Physiol.

[14] Barton PJ, Felkin LE, Birks EJ, Culle ME, Banner NR, Grindle S, Hall JL, Miller LW, Yacoub MH (2005). Myocardial insulin-like growth factor-I gene expression during recovery from heart failure after combined left ventricular assist device and clenbuterol therapy. Circulation.

[15] Pucci A, Zanini C, Granata R, Ghigone R, Iavarone A, Broglio F, Sorrentino P, Bergamasco L, Rinaldi M, Ghigo E (2009). Myocardial insulin-like growth factor-1 and insulin-like growth factor binding protein-3 gene expression in failing hearts harvested from patients undergoing cardiac transplantation. J Heart Lung Transplant.

[16] Arcopinto M, Bobbio E, Bossone E, Perrone-Filardi P, Napoli R, Sacca L, Cittadini A (2013). The GH/IGF-1 axis in chronic heart failure. Endocrin Metab Immun Disord Drug Targets.

[17] Madonna R, Geng YJ, Bolli R, Rokosh G, Ferdinandy P, Patterson C, De Caterina R (2014). Co-activation of nuclear factor-?B and myocardin/serum response factor conveys the hypertrophy signal of high insulin levels in cardiac myoblasts. J Biol Chem.

[18] Shyu KG, Ko WH, Yang WS, Wang BW, Kuan P (2005). Insulin-like growth factor-1 mediates stretch-induced upregulation of myostatin expression in neonatal rat cardiomyocytes. Cardiovasc Res.

[19] Yang W, Zhang Y, Li Y, Wu Z, Zhu D (2007). Myostatin induces cyclin D1 degradation to cause cell cycle arrest through a phosphatidylinositol 3-kinase/AKT/GSK-3 beta pathway and is antagonized by insulin-like growth factor 1. J Biol Chem.

[20] Morissette MR, Cook SA, Buranasombati C, Rosenberg MA, Rosenzweig A (2009). Myostatin inhibits IGF-I-induced myotube hypertrophy through Akt. Am J Physiol Cell Physiol.

[21] Gaussin V, Depre C (2005). Myostatin, the cardiac chalone of insulin-like growth factor-1. Cardiovasc Res.

[22] George I, Bish LT, Kamalakkannan G, Petrilli CM, Oz MC, Naka Y, Sweeney HL, Maybaum S (2010). Myostatin activation in patients with advanced heart failure and after mechanical unloading. Eur J Heart Fail.

[23] Chomczynski P, Sacchi N (1987). Single-step method of RNA isolation by acid guanidinium thiocyanate-phenol-chloroform extraction. Anal Biochem.

[24] Sun Y, Li Y, Luo D, Liao DJ: Pseudogenes as weaknesses of ACTB (Actb) and GAPDH (Gapdh) used as reference genes in reverse transcription and polymerase chain reactions. PLoS One 2012, doi:10.1371/journal.pone.0041659. 10.1371/journal.pone.0041659 PMC3425558 22927912

[25] Torrado M, Iglesias R, Nespereira B, Mikhailov AT. Identification of candidate genes potentially relevant to chamber-specific remodeling in postnatal ventricular myocardium. J Biomed Biotechnol 2010, doi:10.1155/2010/603159. 10.1155/2010/603159 PMC2846348 20368782

[26] Modesti PA, Vanni S, Bertolozzi I, Cecioni I, Lumachi C, Perna AM, Boddi M, Gensini GF (2004). Different growth factor activation in the right and left ventricles in experimental volume overload. Hypertension.

[27] Zungu M, Young ME, Stanley WC, Essop MF (2008). Expression of mitochondrial regulatory genes parallels respiratory capacity and contractile function in a rat model of hypoxia-induced right ventricular hypertrophy. Mol Cell Biochem.

[28] Heineke J, Auger-Messier M, Xu J, Sargent M, York A, Welle S, Molkentin JD (2010). Genetic deletion of myostatin from the heart prevents skeletal muscle atrophy in heart failure. Circulation.

[29] Gruson D, Ahn SA, Ketelslegers JM, Rousseau MF (2011). Increased plasma myostatin in heart failure. Eur J Heart Fail.

[30] Breitbart A, Scharf GM, Duncker D, Widera C, Gottlieb J, Vogel A, Schmidt S, Brandes G, Heuft HG, Lichtinghagen R, Kempf T, Wollert KC, Bauersachs J, Heineke J. Highly specific detection of myostatin prodomain by an immunoradiometric sandwich assay in serum of healthy individuals and patients. Plos One 2013, doi:10.1371/journal.pone.0080454. 10.1371/journal.pone.0080454 PMC3829884 24260393

[31] Lenk K, Erbs S, Höllriegel R, Beck E, Linke A, Gielen S, Winkler SM, Sandri M, Hambrecht R, Schuler G, Adams V (2012). Exercise training leads to a reduction of elevated myostatin levels in patients with chronic heart failure. Eur J Prev Cardiol.

[32] Callis TE, Pandya K, Seok HY, Tang RH, Tatsuguchi M, Huang ZP, Chen JF, Deng Z, Gunn B, Shumate J, Willis MS, Selzman CH, Wang DZ (2009). MicroRNA-208a is a regulator of cardiac hypertrophy and conduction in mice. J Clin Invest.

[33] Satoh M, Minami Y, Takahashi Y, Tabuchi T, Nakamura M (2010). Expression of microRNA-208 is associated with adverse clinical outcomes in human dilated cardiomyopathy. J Card Fail.

[34] Bostjancic E, Zidar N, Stajer D, Glavac D (2010). MicroRNAs miR-1, miR-133a, miR-133b and miR-208 are dysregulated in human myocardial infarction. Cardiology.

[35] Varga ZV, Zvara A, Faragó N, Kocsis GF, Pipicz M, Gáspár R, Bencsik P, Görbe A, Csonka C, Puskás LG, Thum T, Csont T, Ferdinandy P (2014). MicroRNAs associated with ischemia-reperfusion injury and cardioprotection by ischemic pre- and postconditioning: protectomiRs. Am J Physiol Heart Circ Physiol.

[36] Shyu KG, Lu MJ, Wang BW, Sun HY, Chang H (2006). Myostatin expression in ventricular myocardium in a rat model of volume-overload heart failure. Eur J Clin Invest.

[37] McKoy G, Bicknell KA, Patel K, Brooks G (2007). Developmental expression of myostatin in cardiomyocytes and its effect on foetal and neonatal rat cardiomyocyte proliferation. Cardiovasc Res.

[38] Lenk K, Schur R, Linke A, Erbs S, Matsumoto Y, Adams V, Schuler G (2009). Impact of exercise training on myostatin expression in the myocardium and skeletal muscle in a chronic heart failure model. Eur J Heart Fail.

[39] Anderson SB, Goldberg AL, Whitman M (2008). Identification of a novel pool of extracellular pro-myostatin in skeletal muscle. J Biol Chem.

[40] Mendler L, Baka Z, Kovács-Simon A, Dux L (2007). Androgens negatively regulate myostatin expression in an androgen-dependent skeletal muscle. Biochem Biophys Res Commun.

[41] Mendler L, Zádor E, Ver Heyen M, Dux L, Wuytack F (2000). Myostatin levels in regenerating rat muscles and in myogenic cell cultures. J Muscle Res Cell Motil.

[42] Chugh SS, Whitesel S, Turner M, Roberts CT, Nagalla SR (2003). Genetic basis for chamber-specific ventricular phenotypes in the rat infarct model. Cardiovasc Res.

